# Analysis of COVID-19 Infections on a CT Image Using DeepSense Model

**DOI:** 10.3389/fpubh.2020.599550

**Published:** 2020-11-20

**Authors:** Adil Khadidos, Alaa O. Khadidos, Srihari Kannan, Yuvaraj Natarajan, Sachi Nandan Mohanty, Georgios Tsaramirsis

**Affiliations:** ^1^Department of Information Technology, Faculty of Computing and Information Technology, King Abdulaziz University, Jeddah, Saudi Arabia; ^2^Department of Information Systems, Faculty of Computing and Information Technology, King Abdulaziz University, Jeddah, Saudi Arabia; ^3^Department of Computer Science and Engineering, SNS College of Engineering, Coimbatore, India; ^4^Research and Development, Information Communication Technology Academy, Chennai, India; ^5^Department of Computer Science and Engineering, Institute of Chartered Financial Analysts of India Foundation of Higher Education, Hyderabad, India; ^6^Higher Colleges of Technology, Women's College, Abu Dhabi, United Arab Emirates

**Keywords:** DeepSense, artificial intelligence, convolutional neural network, CT images, prediction, COVID-19

## Abstract

In this paper, a data mining model on a hybrid deep learning framework is designed to diagnose the medical conditions of patients infected with the coronavirus disease 2019 (COVID-19) virus. The hybrid deep learning model is designed as a combination of convolutional neural network (CNN) and recurrent neural network (RNN) and named as DeepSense method. It is designed as a series of layers to extract and classify the related features of COVID-19 infections from the lungs. The computerized tomography image is used as an input data, and hence, the classifier is designed to ease the process of classification on learning the multidimensional input data using the Expert Hidden layers. The validation of the model is conducted against the medical image datasets to predict the infections using deep learning classifiers. The results show that the DeepSense classifier offers accuracy in an improved manner than the conventional deep and machine learning classifiers. The proposed method is validated against three different datasets, where the training data are compared with 70%, 80%, and 90% training data. It specifically provides the quality of the diagnostic method adopted for the prediction of COVID-19 infections in a patient.

## Introduction

The novel coronavirus disease 2019 (COVID-19) is a pandemic outbreak (1). COVID-19 patients are classified essentially based on computerized tomography (CT) lung images, and it is used widely for testing. The healthcare institutions fitted with CT scans help in the process of image acquisition and classification of CT images at a faster rate. However, the need for an expert medical practitioner is hence required for the verification of the final results, which increases the time of computation (2). On the other hand, the supervised learning models (3–10) can be utilized for classifying the patients from the CT images.

Infections based on CT images are not classified using very little unattended methods (11–24). We have developed a model that mainly includes supervised and unsupervised learning models in order to improve the classification process. The aim is to classify the infected patients automatically based on their CT images.

In this paper, a DeepSense algorithm is utilized to diagnose COVID-19 infections among the medical community. The deep learning method is designed as a combination of convolutional neural network (CNN) and recurrent neural network (RNN) that reduces the classifier burden on optimal classification of the multidimensional data features.

The main contribution of the work includes the following:

(a) The authors develop a combined CNN and RNN to classify the medical image datasets.
(b) The experimental results are conducted to measure the correctness in terms of its accuracy, precision, and recall values against artificial neural network (ANN), feedforward neural network (FFNN), back propagation neural network (BPNN), deep neural network (DNN), and RNN.

The outline of the paper is presented as follows: The *Methods* section provides the details of the ensemble classifier. The *DeepSense Model* section evaluates the entire work. The *Results and Discussions* section concludes the work with future enhancement.

## Methods

**Figure 1:**
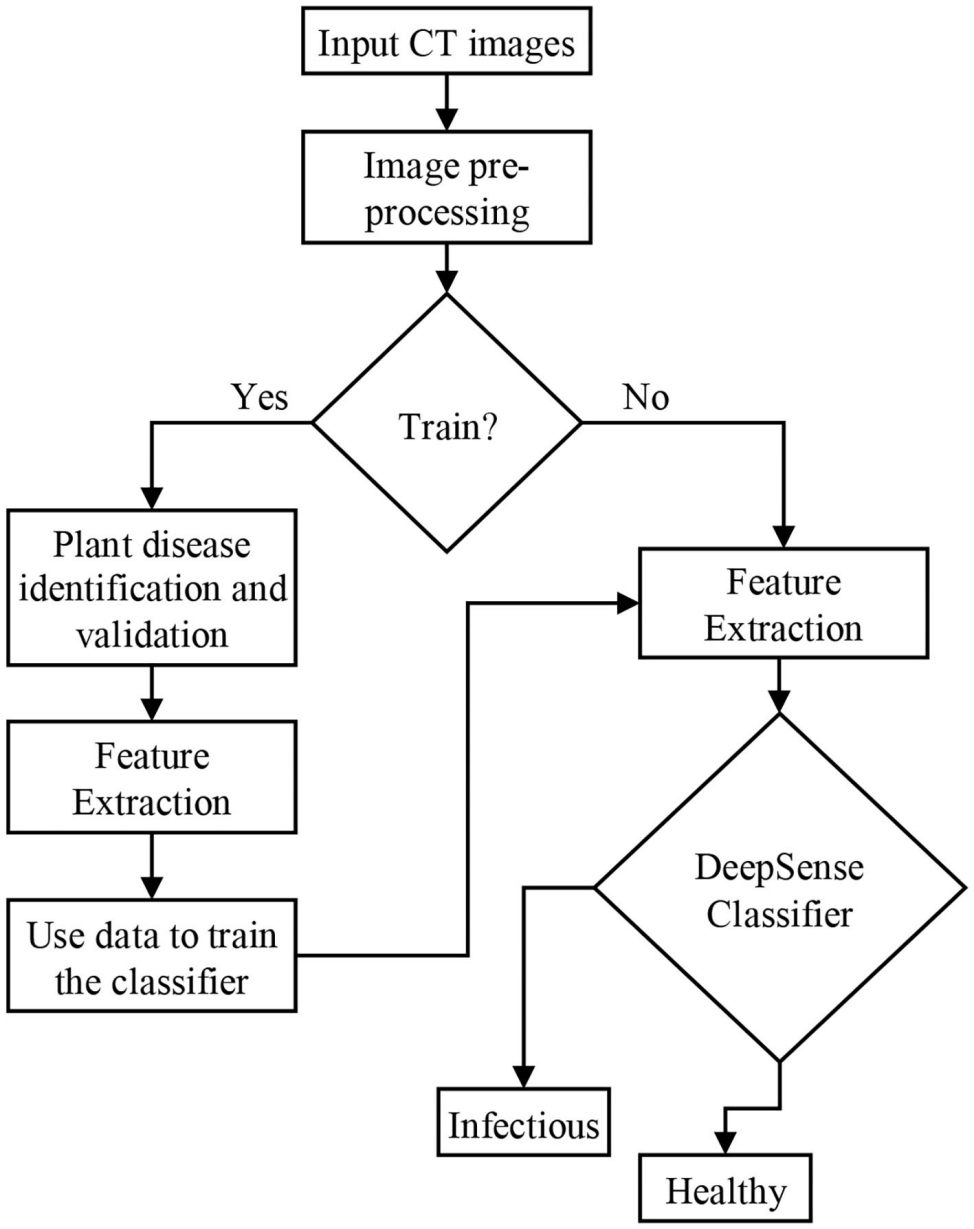
Proposed model for classification.

The deep learning model namely DeepSense algorithm is a combination of CNN and RNN designed to improve the performance of the classification accuracy. DeepSense learning is regarded as a module for accurate predictions of lung infections caused by the COVID-19 virus. Figure 1 shows the architecture of the proposed classification model using the DeepSense algorithm.

## Deepsense Model

**Figure 2:**
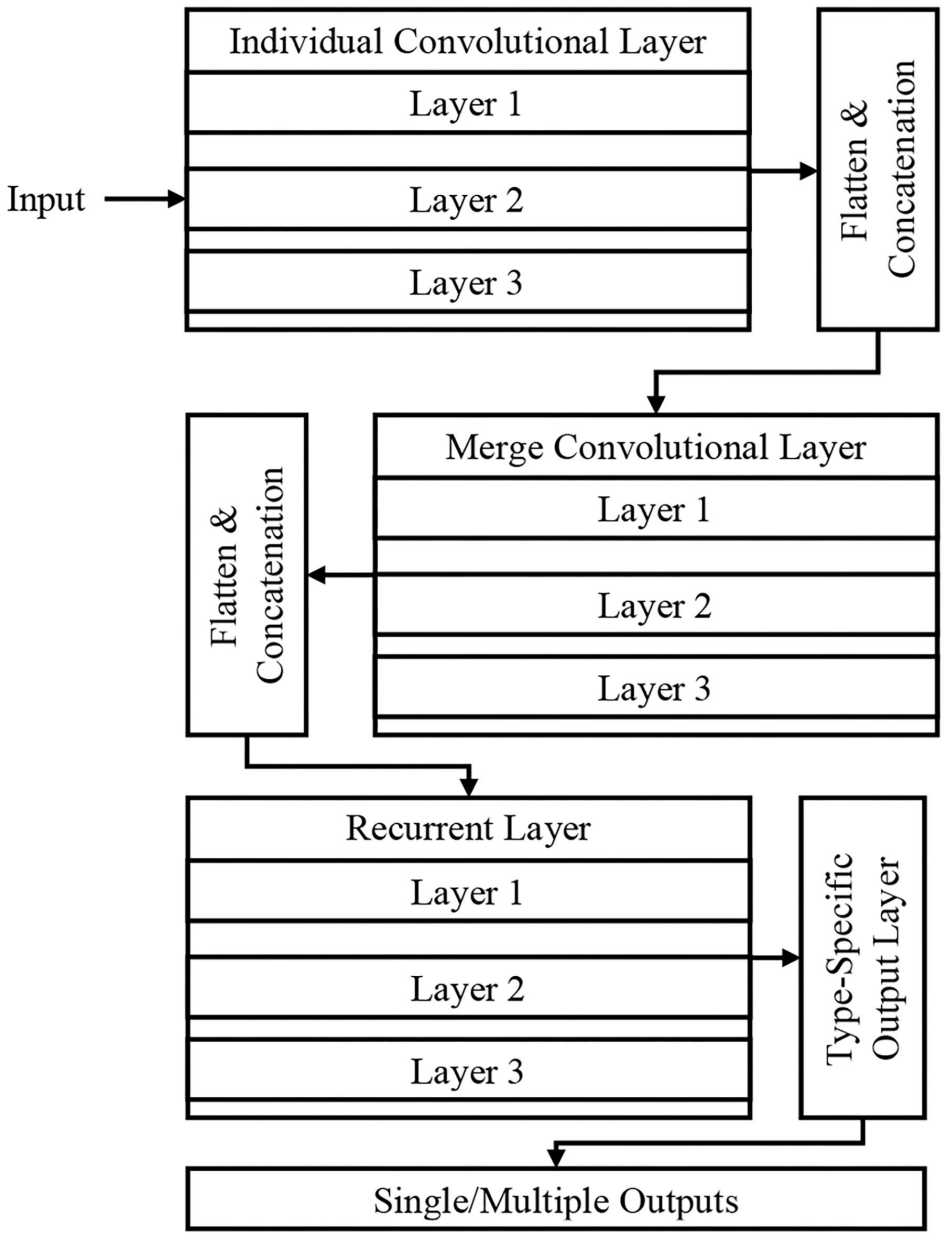
Proposed DeepSense deep neural network (DNN) architecture.

Figure 2 shows the DeepSense DNN (25) model that has three components, including convolutional, recurrent, and output layer that are stacked upon one another. The convolutional and recurrent layers are regarded as the significant building blocks (Figure 1), and the output layer is considered as a specific layer that classifies the images. The DeepSense DNN model is designed for the classification of input CT images for COVID-19-related infections.

The DeepSense network avoids gradient exploding and improves the rate of convergence using residual learning, adjustable learning rate, and gradient clipping that helps in optimizing the process of training.

The features are extracted through the DeepSense model that increases the reconstruction accuracy and reduces the time of training. Such optimization helps in obtaining the rich text information, and it has better ability for classification.

### Convolutional Layers

The convolutional layers have three different parts that include individual convolutional subnets for input from CT device *X*(*k*), where *k* is the number of CT device. The other subnets include a merged convolutional subnet for *K* convolutional subnets' outputs.

For a time interval *t*, the matrix *X*(*k*) is used as an input to the DNN architecture that extracts the relationship of *X*(*k,t*), which includes the relationships lying inside the frequency domain. The sensor measurement interactions include entire dimension, where the frequency domain usually has several local patterns. These interactions are studied using 2D filters and produces the output *X*(*k,1,t*) based on the local patterns and dimensions in frequency domain. The high-level relationships are learned hierarchically using the application of a 1D filter. The matrix is then flattened into a vector, and they are concatenated to produce the input for RNN layers. The activation function in the convolutional layer is a rectified linear unit (ReLU) function, and batch normalization eliminates the internal covariate shift.

### Recurrent Layers

The RNN architecture learns the needed features having long-term dependencies (long paths). The study uses Gated Recurrent Unit (GRU) on long and short path selection to reduce well the network complexity. A set of three layers stacked in GRU is used in this paper that uses time flow that runs the stacked GRU incrementally for faster input data processing. The recurrent layer outputs vector series {*x*(*r,t*)} where *t* = 1,2,···, *T* for the process of classification at the output layer.

### Output Layer

For the purpose of classification, {*x*(*r,t*)} is selected as the feature vector, and this layer converts the vector of variable length into fixed length. The final feature is generated by averaging the features over a specific time interval based on long or short paths, *x*(*r*) $x\left(r\right)=\frac{\overset{T}{\underset{t=1}{\sum }}x\left(t,r\right)}{T}$. Finally the probability of predicted category is generated by feeding the averaging features into the softmax layer.

### Type-Specific Layer

For the customization of the DeepSense layer to operate the process of classification, we specifically use the following process:

Step 1: Identify the input image
Step 2: Preprocessing input image for temporal and spectral noise
Step 3: Extract the features related to COVID-19 infections
Step 4: Apply DeepSense classifier for optimal classifier.

## Results and Discussions

This section provides the results of comparison between the machine/deep learning classifiers for predicting COVID-19 infections using IEEE8023 (26), COVID-CT-Dataset (27), and COVID-19 Open Research Dataset Challenge (CORD-19) (28) datasets.

IEEE8023 has the image collection from various sources including COVID-19 or viral and bacterial pneumonias in the form of CT images. COVID-CT has 349 COVID-19 CT images from 216 patients and 463 non-COVID-19 CTs. CORD-19 has collected the CT image resources from 52,000 scholarly articles.

The study is experimented using a 10-fold cross validation, which is tested with all these three base classifiers.

### Experiment

The performance measures for evaluating the DeepSense classifier is estimated against various metrics: accuracy, geometric mean (G-mean), F-measure, precision, percentage error, specificity, and sensitivity.

Accuracy for optimal classification is given below:

(1) $$\begin{array}{r} Accuracy=\frac{TP+TN}{TP+TN+FP+FN} \end{array}$$

where:

*TP* is defined as the true positive
*TN* is defined as the true negative
*FP* is defined as the false positive
*FN* is defined as the false negative

F-measure of the DeepSense classifier is defined as follows:

(2) $$\begin{array}{r} F-measure=\frac{2TP}{2TP+FP+FN} \end{array}$$

G-mean of the DeepSense classifier is defined as follows:

(3) $$\begin{array}{r} G-mean=\sqrt{\frac{TP}{TP+FN}\times \frac{TN}{TN+FP}} \end{array}$$

Mean absolute percentage error (MAPE) of the DeepSense classifier is defined as follows:

(4) $$\begin{array}{r} MAPE=\frac{100}{n}\overset{n}{\underset{t=1}{\sum }}|\frac{A_{t}-F_{t}}{A_{t}}| \end{array}$$

Where

*A*_*t*_ is defined as the actual class
*F*_*t*_ is defined as the predicted class, and
*n* is defined as the fitted points

Sensitivity of the DeepSense is defined as:

(5) $$\begin{array}{r} Sensitivity=\frac{TP}{TP+FN} \end{array}$$

Specificity of the DeepSense is defined as:

(6) $$\begin{array}{r} Specificity=\frac{TN}{TN+FP} \end{array}$$

### Analysis

**Figure 3:**
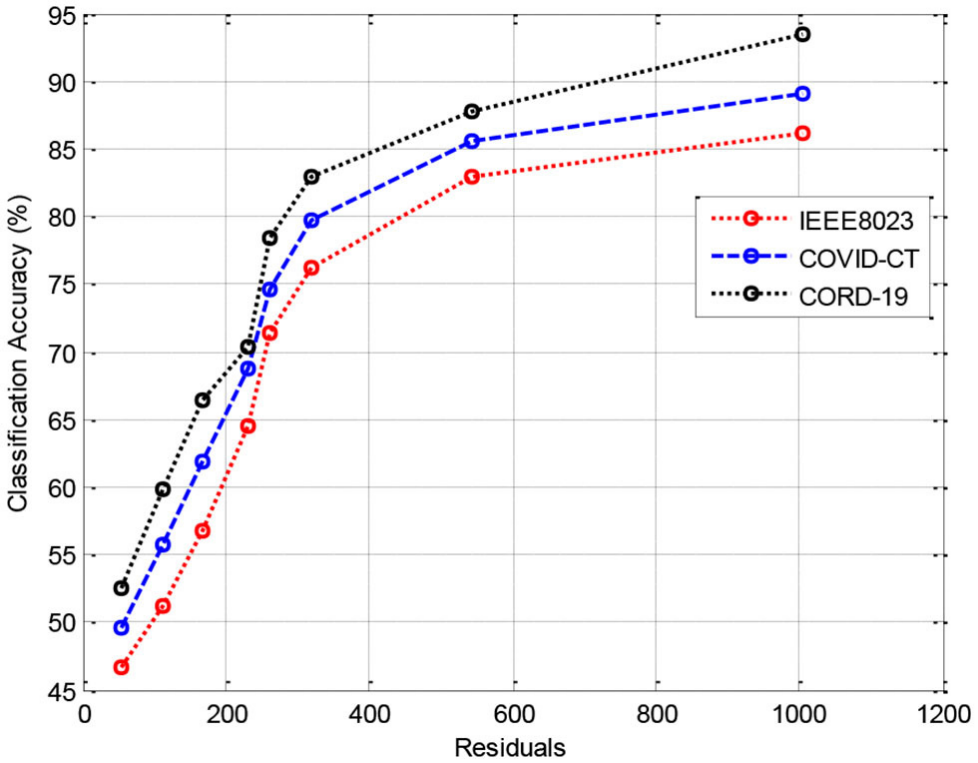
Results of classification accuracy during training with 70% training data.

**Figure 4:**
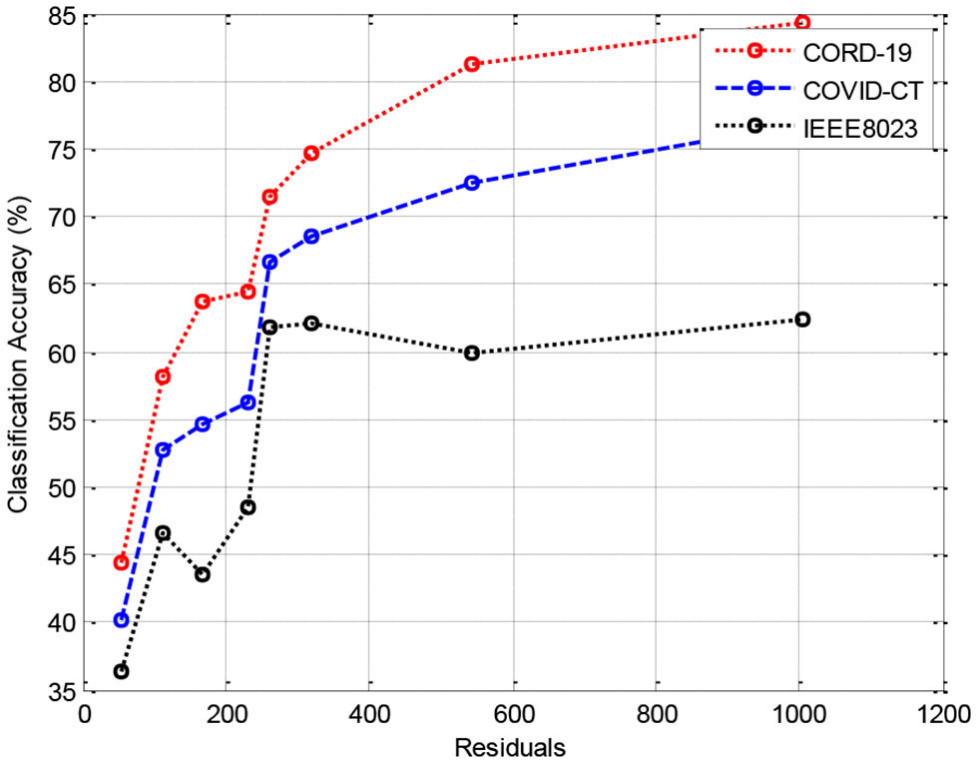
Results of classification accuracy during training with 80% training data.

**Figure 5:**
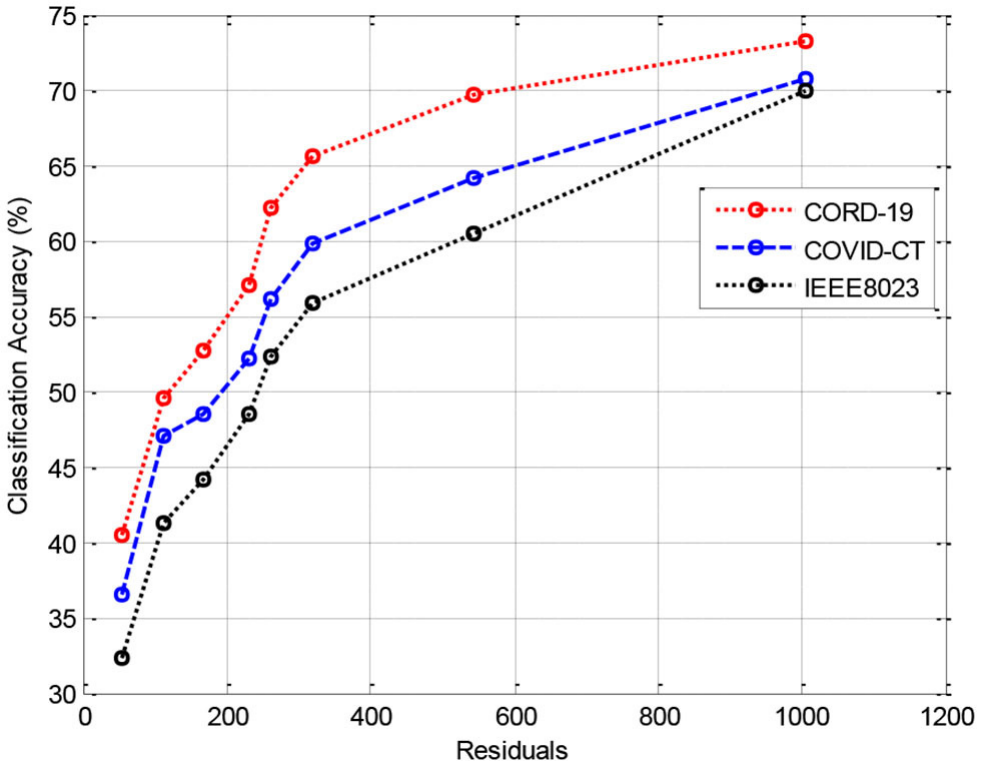
Results of classification accuracy during training with 90% training data.

In this section, we provide the results of various meta-ensemble classifiers that include FFNN (29), ANN (25), DNN (30), BPNN (31), and RNN (32). The proposed method is validated against three different datasets, where the training data are compared with 70% (Figure 3), 80% (Figure 4), and 90% (Figure 5) training data.

Figure 3 shows the results of classification accuracy of CORD-19 datasets for all residuals are higher, and with increasing residuals, the accuracy increases. Same is the case for other training sets; however, with 80% datasets, the accuracy is fluctuating due to the extraction of on-optimal features from IEEE8023 datasets.

Tables 1, 4, 7 provide the results of statistical parameters on predicting COVID-19 infections over 70, 80, and 90% training data over IEEE8023 datasets.

**Table 1:** Results of statistical parameters for IEEE8023 with 70% training data on 1,000 images.

**Statistical parameters**	**ANN**	**FFNN**	**BPNN**	**DNN**	**RNN**	**DeepSense**
Accuracy	55.67145	55.97152	58.06198	58.32304	59.68335	80.475
F-measure	38.39159	40.49205	51.72857	51.8886	54.26013	83.65671
G-mean	72.54022	72.77127	74.27161	74.31162	74.72171	85.57814
MAPE	28.32533	25.38368	23.98336	21.40179	20.82166	16.1186
Sensitivity	61.74481	65.25659	73.16136	85.54813	86.20828	96.25452
Specificity	74.18159	74.37163	77.88342	77.90342	79.27473	80.11492

*ANN, artificial neural network; BPNN, back propagation neural network; DNN, deep neural network; FFNN, feedforward neural network; MAPE, mean absolute percentage error; RNN, recurrent neural network*.

Tables 2, 5, 8 provide the results of statistical parameters on predicting COVID-19 infections over 70, 80, and 90% training data over COVID-CT datasets.

**Table 2:** Results of statistical parameters for COVID-CT with 70% training data on 1,000 images.

**Statistical parameters**	**ANN**	**FFNN**	**BPNN**	**DNN**	**RNN**	**DeepSense**
Accuracy	56.26158	58.87317	61.23469	62.605	65.84672	84.68794
F-measure	66.74693	66.79694	67.68814	68.80839	73.84151	79.40476
G-mean	43.50373	56.43162	59.4633	44.705	76.09302	85.98823
MAPE	19.37033	16.69873	16.60871	11.75563	10.42533	9.275074
Sensitivity	76.23305	78.91465	79.00467	83.84675	85.18805	86.33831
Specificity	73.39141	76.27306	77.21327	80.37497	82.37642	84.46789

*ANN, artificial neural network; BPNN, back propagation neural network; COVID-19, coronavirus disease 2019; DNN, deep neural network; FFNN, feedforward neural network; MAPE, mean absolute percentage error; RNN, recurrent neural network*.

Tables 3, 6, 9 provide the results of statistical parameters on predicting COVID-19 infections over 70, 80, and 90% training data over CORD-19 datasets.

**Table 3:** Results of statistical parameters for CORD-19 with 70% training data on 1,000 images.

**Statistical parameters**	**ANN**	**FFNN**	**BPNN**	**DNN**	**RNN**	**DeepSense**
Accuracy	59.07321	65.85673	68.8784	74.09157	77.93343	82.41643
F-measure	69.68858	69.93964	70.10968	70.28972	74.85174	80.39498
G-mean	69.98965	70.2197	71.94009	74.00155	76.4631	79.21471
MAPE	68.11823	64.47642	57.75191	39.63186	36.77022	34.91881
Sensitivity	77.52334	71.14991	71.87007	73.64147	73.84151	80.69505
Specificity	70.39974	72.28016	75.35185	80.58502	81.89631	82.30641

*ANN, artificial neural network; BPNN, back propagation neural network; CORD-19, COVID-19 Open Research Dataset Challenge; COVID-19, coronavirus disease 2019; DNN, deep neural network; FFNN, feedforward neural network; MAPE, mean absolute percentage error; RNN, recurrent neural network*.

**Table 4:** Results of statistical parameters for IEEE8023 with 80% training data on 1,000 images.

**Statistical parameters**	**ANN**	**FFNN**	**BPNN**	**DNN**	**RNN**	**DeepSense**
Accuracy	96.46457	97.18473	97.21474	97.29476	97.30476	97.43479
F-measure	52.36871	69.72859	70.04966	72.93131	76.16303	79.36475
G-mean	81.88631	82.7365	84.35786	85.91821	90.96134	92.48168
MAPE	26.90502	25.51371	22.74209	20.07049	10.60537	90.12115
Sensitivity	68.69836	70.08967	72.86129	75.54189	84.99801	88.59981
Specificity	96.53459	97.32476	97.52481	97.60483	97.62483	97.68484

*ANN, artificial neural network; BPNN, back propagation neural network; DNN, deep neural network; FFNN, feedforward neural network; MAPE, mean absolute percentage error; RNN, recurrent neural network*.

**Table 5:** Results of statistical parameters for COVID-CT with 80% training data on 1,000 images.

**Statistical parameters**	**ANN**	**FFNN**	**BPNN**	**DNN**	**RNN**	**DeepSense**
Accuracy	97.73486	97.75486	97.77486	97.77486	97.78487	97.78487
F-measure	89.19995	90.63127	90.7813	91.29141	91.50146	92.1416
G-mean	93.27286	96.67462	97.22474	97.52481	97.66484	97.66484
MAPE	86.64838	27.01504	20.25053	9.275074	54.63022	21.0017
Sensitivity	88.94989	95.58337	96.68462	97.26475	97.54481	97.55482
Specificity	96.76464	96.78464	96.78464	96.78464	96.78464	97.42479

*ANN, artificial neural network; BPNN, back propagation neural network; COVID-19, coronavirus disease 2019; DNN, deep neural network; FFNN, feedforward neural network; MAPE, mean absolute percentage error; RNN, recurrent neural network*.

**Table 6:** Results of statistical parameters for CORD-19 with 80% training data on 1,000 images.

**Statistical parameters**	**ANN**	**FFNN**	**BPNN**	**DNN**	**RNN**	**DeepSense**
Accuracy	93.97301	94.12305	94.20307	94.25308	94.43312	94.44312
F-measure	58.25303	60.04343	60.3835	60.84361	62.37495	62.51498
G-mean	79.1447	79.65481	80.18493	80.41498	81.52623	81.98633
MAPE	29.90669	29.17552	28.28533	27.93525	26.10384	25.28365
Sensitivity	65.69669	66.42685	67.31805	67.67813	69.49854	70.32973
Specificity	95.2533	95.41334	95.42334	95.47335	95.51336	95.56337

*ANN, artificial neural network; BPNN, back propagation neural network; CORD-19, COVID-19 Open Research Dataset Challenge; COVID-19, coronavirus disease 2019; DNN, deep neural network; FFNN, feedforward neural network; MAPE, mean absolute percentage error; RNN, recurrent neural network*.

**Table 7:** Results of statistical parameters for IEEE8023 with 90% training data on 1,000 images.

**Statistical parameters**	**ANN**	**FFNN**	**BPNN**	**DNN**	**RNN**	**DeepSense**
Accuracy	95.91445	95.93445	95.94446	96.03448	96.05448	96.11449
F-measure	77.3833	77.51333	78.03345	79.11469	79.79484	80.08491
G-mean	79.43476	79.67482	79.94488	80.9451	81.26517	81.45622
MAPE	31.14697	30.78688	30.26677	28.65541	28.1553	27.83522
Sensitivity	64.45641	64.81649	65.33661	66.94797	67.44808	67.76815
Specificity	94.72318	94.7832	94.8232	96.05448	96.46457	96.81465

*ANN, artificial neural network; BPNN, back propagation neural network; DNN, deep neural network; FFNN, feedforward neural network; MAPE, mean absolute percentage error; RNN, recurrent neural network*.

**Table 8:** Results of statistical parameters for COVID-CT with 90% training data on 1,000 images classifier.

**Statistical parameters**	**ANN**	**FFNN**	**BPNN**	**DNN**	**RNN**	**DeepSense**
Accuracy	97.37478	97.37478	97.45479	97.45479	97.4748	97.52481
F-measure	85.88821	86.00823	87.94967	87.97967	89.33998	89.34998
G-mean	94.03303	94.03303	94.40311	94.47313	94.8032	94.84321
MAPE	70.79983	70.61979	62.72503	61.39473	54.04008	53.35993
Sensitivity	90.53124	90.53124	91.34143	91.47146	92.21162	92.28164
Specificity	97.4648	97.4748	97.56482	97.56482	97.65484	97.65484

*ANN, artificial neural network; BPNN, back propagation neural network; COVID-19, coronavirus disease 2019; DNN, deep neural network; FFNN, feedforward neural network; MAPE, mean absolute percentage error; RNN, recurrent neural network*.

**Table 9:** Results of statistical parameters for CORD-19 with 90% training data on 1,000 images.

**Statistical parameters**	**ANN**	**FFNN**	**BPNN**	**DNN**	**RNN**	**DeepSense**
Accuracy	97.44379	97.44379	97.52381	97.52381	97.54381	97.59382
F-measure	85.94922	86.06925	88.01168	88.04169	89.40299	89.41399
G-mean	94.09904	94.09904	94.47013	94.54014	94.87022	94.91022
MAPE	70.84984	70.6698	62.77004	61.43874	54.07909	53.39794
Sensitivity	90.59526	90.59526	91.40644	91.53647	92.27664	92.34765
Specificity	97.53381	97.54381	97.63383	97.63383	97.72385	97.72385

*ANN, artificial neural network; BPNN, back propagation neural network; CORD-19, COVID-19 Open Research Dataset Challenge; COVID-19, coronavirus disease 2019; DNN, deep neural network; FFNN, feedforward neural network; MAPE, mean absolute percentage error; RNN, recurrent neural network*.

### Evaluation Criteria

The simulation results show that the DeepSense classifier has higher classification accuracy than the existing meta-ensemble classifiers. In addition, the CORD-19 datasets offer optimum selection of features to increase classification accuracy by 90% training data over 80 or 90%. The other measurements are optimal for CORD-19 than the other selection tools. Furthermore, MAPE is less than the other methods in the deep learning model.

The result shows that the CORD-19 datasets are more accurate than RNN and DNN. The results also show that the classification accuracy with IEEE8023 as a functional selection tool decreases at some point as the number of residues increases compared to COVID-CT and CORD-19. The class of infections is therefore accurately determined with the proposed classification.

## Conclusions and Future Work

In this paper, a DeepSense algorithm is designed for the classification of COVID-19 infections. The DeepSense algorithm helps in optimal classification of multidimensional features from CT images. The classifier combined with hybrid deep learning classifier, namely, CNN and RNN, helps in improving the prediction of events from a medical image. The extraction of optimal features from the feature extraction model helps the classifier to optimally detect whether the patient is infected or not. The experimental results show that the proposed method has higher accuracy than the other methods. In the future, the model can be designed with an ensemble data model to classify the highly rated multidimensional dataset.

## Data Availability Statement

The original contributions presented in the study are included in the article/supplementary materials, further inquiries can be directed to the corresponding author/s.

## Author Contributions

AK: visualization and investigation. AOK: data curation, software, and validation. SK: methodology, data curation, review and editing, and supervision. YN: conceptualization, methodology, writing original draft, software, and data curation. SM: software and validation. GT: writing—review and editing and supervision.

## Conflict of Interest

The authors declare that the research was conducted in the absence of any commercial or financial relationships that could be construed as a potential conflict of interest.
